# Rhythms of Core Clock Genes and Spontaneous Locomotor Activity in Post-*Status Epilepticus* Model of Mesial Temporal Lobe Epilepsy

**DOI:** 10.3389/fneur.2018.00632

**Published:** 2018-08-02

**Authors:** Heloisa de Carvalho Matos, Bruna Del Vechio Koike, Wanessa dos Santos Pereira, Tiago G. de Andrade, Olagide W. Castro, Marcelo Duzzioni, Maheedhar Kodali, Joao P. Leite, Ashok K. Shetty, Daniel L. G. Gitaí

**Affiliations:** ^1^Department of Cellular and Molecular Biology, Institute of Biological Sciences and Health, Federal University of Alagoas, Maceio, Brazil; ^2^Laboratory of Molecular Chronobiology, Federal University of Alagoas, Arapiraca, Brazil; ^3^Department of Physiology and Pharmacology, Institute of Biological Sciences and Health, Federal University of Alagoas, Maceio, Brazil; ^4^Institute for Regenerative Medicine, Department of Molecular and Cellular Medicine, Texas A&M Health Science Center College of Medicine, College Station, TX, United States; ^5^Division of Neurology, Department of Neurosciences and Behavioral Sciences, Ribeirão Preto School of Medicine, University of São Paulo, Ribeirão Preto, Brazil; ^6^Faculty of Medicine, Federal University of Alagoas, Maceio, Brazil

**Keywords:** clock genes, epilepsy, seizure, spontaneous locomotor activity, circadian rhythm

## Abstract

The interaction of Mesial Temporal Lobe Epilepsy (mTLE) with the circadian system control is apparent from an oscillatory pattern of limbic seizures, daytime's effect on seizure onset and the efficacy of antiepileptic drugs. Moreover, seizures *per se* can interfere with the biological rhythm output, including circadian oscillation of body temperature, locomotor activity, EEG pattern as well as the transcriptome. However, the molecular mechanisms underlying this cross-talk remain unclear. In this study, we systematically evaluated the temporal expression of seven core circadian transcripts (*Bmal1, Clock, Cry1, Cry2, Per1, Per2*, and *Per3*) and the spontaneous locomotor activity (SLA) in post-*status epilepticus* (SE) model of mTLE. Twenty-four hour oscillating SLA remained intact in post-SE groups although the circadian phase and the amount and intensity of activity were changed in early post-SE and epileptic phases. The acrophase of the SLA rhythm was delayed during epileptogenesis, a fragmented 24 h rhythmicity and extended active phase length appeared in the epileptic phase. The temporal expression of circadian transcripts *Bmal1, Cry1, Cry2, Per1, Per2*, and *Per3* was also substantially altered. The oscillatory expression of *Bmal1* was maintained in rats imperiled to SE, but with lower amplitude (A = 0.2) and an advanced acrophase in the epileptic phase. The diurnal rhythm of *Cry1* and *Cry2* was absent in the early post-SE but was recovered in the epileptic phase. *Per1* and *Per2* rhythmic expression were disrupted in post-SE groups while *Per3* presented an arrhythmic profile in the epileptic phase, only. The expression of *Clock* did not display rhythmic pattern in any condition. These oscillating patterns of core clock genes may contribute to hippocampal 24 h cycling and, consequently to seizure periodicity. Furthermore, by using a pool of samples collected at 6 different Zeitgeber Times (ZT), we found that all clock transcripts were significantly dysregulated after SE induction, except *Per3* and *Per2*. Collectively, altered SLA rhythm in early post-SE and epileptic phases implies a possible role for seizure as a nonphotic cue, which is likely linked to activation of hippocampal–accumbens pathway. On the other hand, altered temporal expression of the clock genes after SE suggests their involvement in the MTLE.

## Introduction

The circadian timing system generates 24 h oscillations of metabolic, physiologic and behavioral functions as an adaptive response to diurnal environmental changes (1, 2). In mammals, this temporal program is systemically regulated by cells in the suprachiasmatic nucleus (SCN) of the hypothalamus. SCN is the master pacemaker, which integrates environmental time cues (e.g., light/dark cycle) for the necessary synchronization of different tissues in the body (peripheral oscillators) (3, 4). These circadian oscillations are driven by a network of transcription/translation-based interlocking feedback loops, involving a CLOCK/BMAL1 and Period (PER1, PER2, and PER3)/Cryptochrome (CRY1 and CRY2) protein complexes (5–7). The CLOCK and BMAL1 form a heterocomplex that induces the transcription of *Per* and *Cry* genes (positive feedback loop). On the other hand, PERs and CRYs form complexes that suppress the activity of CLOCK and BMAL1 and their expression (negative feedback loop), allowing the cycle to repeat (7, 8). This self-oscillating molecular machinery regulates the expression of several clock-controlled genes in different tissues, which is critical for circadian outputs (9, 10). Multiple studies have shown that circadian disruption might be the cause or consequence of various human diseases (11–19).

Mesial Temporal Lobe Epilepsy (mTLE) is a chronic disease characterized by spontaneous and recurrent partial seizures (SRS) elicited by an excessive and overly synchronized neuronal activity in limbic regions of the brain. An intriguing well-documented feature in this condition is the occurrence of seizures. Despite being unpredictable, seizure episodes display a circadian rhythm (20). In fact, it has been shown in mTLE patients, that seizures present a 24 h non-random pattern of distribution with a unimodal or bimodal distribution (20–22). Similarly, experimental models of mTLE exhibit a diurnal oscillation pattern for the occurrence of SRS (23–30), with an exception (31). These data suggest that the limbic seizures are modulated by a circadian regulatory system.

The pathogenesis of mTLE is a complex process having a close relationship with the circadian system. Prior to the onset of SRS, the brain undergoes series of progressive structural and cellular alterations and reorganization elicited by an initial precipitating injury, such as *Status Epilepticus* (SE). The changes, collectively referred to as epileptogenic processes, comprise neuron loss, aberrant neurogenesis, gliosis, axonal damage, dendritic plasticity, altered gene expression, and inflammation. Such changes occur prominently in the hippocampal formation (32–38), a region of the brain identified as an important peripheral oscillator in mammalian (39–44). Nonetheless, the extent to which circadian rhythms and epileptogenesis influence each other remains unclear though studies point to the presence of bidirectional interaction (13, 27, 45–48). On the one hand, some studies imply that the time of the day has an effect on the threshold for seizure induction in different experimental models (49–53). For example, in pilocarpine (PILO) induced SE, both shorter latency to seizures and severe seizures were associated with the induction of SE in daytimes vis-à-vis the dark period. Moreover, circadian disruption through knockdown of *Bmal1* or clock-controlled genes leads to a reduced seizure threshold (54, 55). On the other hand, epileptogenic processes can alter circadian biological rhythms. It has been reported in experimental models of mTLE that changes in the diurnal rhythms of spontaneous locomotor activity (SLA) and temperature body occur a few weeks after SE induction or/and after SRS onset (27, 56–58). Continuous 24/7 video/telemetric hippocampal EEG recordings in a PILO-induced SE have revealed that SE induces a transient suppression of the physiologic circadian EEG pattern, which correlates with less severe seizures during this period. Remarkably, SRS ensue after the stabilization of the circadian rhythm and start to emerge in clusters or a more severe manner (59).

However, molecular mechanisms involved in the interaction between epileptogenesis and the circadian system remains unclear. Partly, this is due to a lack of studies on the rhythmic expression of clock genes in epileptic phases. A recent study, using large-scale approaches, demonstrated a diurnal molecular landscape in the hippocampus of epileptic mice [(60), preprint publication]. However, the potential changes in clock genes across different phases of epileptogenesis have not been investigated. It is also unknown how does circadian gene expression relate to induction of spontaneous seizures and epilepsy. In our previous study, we analyzed the temporal profiling of clock genes mRNA levels in the hippocampus of naïve rats (61). Here, we address this issue by studying these genes in a post-SE model of mTLE, a prototype showing similar epileptogenic and histopathological processes as human mTLE (62). First, we evaluated the effects of SE on diurnal rhythms of SLA in rats during the early post-SE and epileptic periods. Next, we examined the temporal expression of *Clock, Bmal1, Cry1, Cry2, Per1, Per2*, and *Per3* mRNA levels in the hippocampus of naïve rats and rats subjected to SE in early post-SE and epileptic phases. Finally, the gene expression levels were investigated by RT-qPCR in the hippocampus of experimental and control animals.

## Materials and methods

### Animals and experimental groups

**Figure 1:**
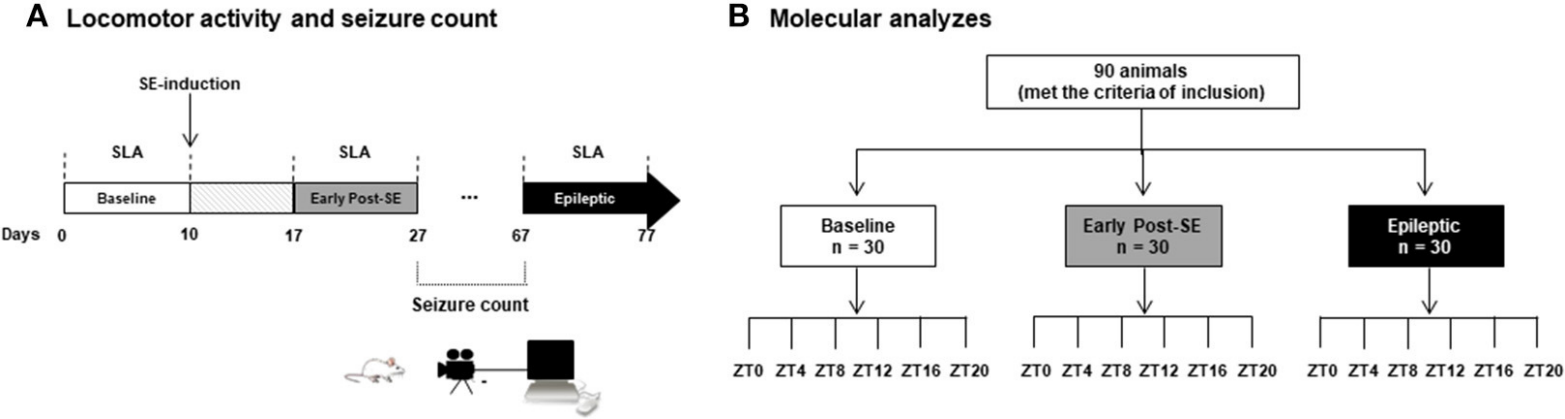
Experimental design. **(A)** Rats assigned to the SLA experiments (*n* = 10) were placed on the rack equipped with sensors in order to record the spontaneous locomotor activity. The basal activity was considered the recording of 10 days in free movement, what we called baseline (white bar). At 10th day, these rats received the pilocarpine injection to promote the status epilepticus (SE). After a week, the animals were in the early post-SE phase, and the SLA was recorded for 10 days (from day 17 to 27, gray bar), and they are considered as early post-SE group. The animals were transferred to the video recording chamber, and the seizure observation was done every day from 10 a.m. to 6 p.m. All animals that presented two or more stage 3 seizures (Racine scale) were considered for the epileptic group. These rats were then re-placed at the SLA recording chamber, and the SLA was recorded for 10 days (black bar). **(B)** Rats belonging to molecular analyses (*n* = 90) were euthanized every 4 h during a 24 h period (five animals per time point) at the ZT 0, 4, 8, 12, 16, and 20 for each group. All these animals were euthanized by decapitation using a guillotine within 20 min of the each ZT. For ZT12, ZT16, and ZT20, the euthanasia was done in the dim red light. SE, *Status Epilepticus;* SLA, Spontaneous Locomotor Activity; ZT, Zeitgeber time units.

Experiments included 100 male adult Wistar rats (300–400 g) from the main breeding stock of the Federal University of Alagoas. Ten rats were designated for SLA experiments, and 90 rats were used for molecular analysis (Figure 1). Rats were 192–206 days-old and kept at 22 ± 2°C in groups of five per cage with free access to food and water. The animals were under a 12 h light and 12 h dark regimen, which was divided into 24 h Zeitgeber time units (ZT), where ZT0 is when the light is turned on (6 a.m.) and ZT12 when the light is turned off (6 p.m.). The rats assigned to SLA experiments (*n* = 10) were the same recorded during the baseline. These comprise the naïve control group, the early post-SE group (i.e., 17 days after SE-induction), and the epileptic group (Figure 1A). Rats belonging to molecular analyses (*n* = 90) were euthanized every 4 h during a 24 h period (five animals per time point) at the ZT 0, 4, 8, 12, 16 and 20 for each group. These animals were euthanized by decapitation using a guillotine within 20 min of the each ZT. For ZT12, ZT16, and ZT20, the euthanasia was done in dim red light (Figure 1B). All animal experiments were performed per a protocol approved by the Research Ethics Committee of the Federal University of Alagoas (Permit number: 12/2014) and were consistent with the International guidelines of the ethical use of animals, such as those from the Society for Neuroscience. Animal health was monitored throughout the experimental period as described previously (61). All efforts were made to reduce the number of animals used and to avoid any unnecessary suffering. No animals presented clinical/behavioral signs of pain or unexpected distress used as humane endpoint criteria for euthanasia. We have followed the standard biosecurity.

### Post-SE model of MTLE

The SE induction and evaluation were performed as detailed elsewhere (63). Briefly, rats were injected intraperitoneally (i.p.) with lithium chloride (127 mg/kg, Sigma) followed by PILO (30 mg/kg, Sigma) after 18 h. To counteract the peripheral cholinergic effects, scopolamine butyl-bromide (1 mg/kg, Sigma) was administered 30 min before the administration of PILO. Animals were kept in SE for 90 min before seizure interruption with an injection of diazepam (5 mg/kg; i.p.). For the molecular analysis, from post-SE day 3, animals were individually placed in acrylic cages, and their behavior was videotaped for ~8 h per day over 11 weeks. All videos were analyzed by two independent observers, and the severity score of SRS was classified as per Racine scale, which is based on five stages: stage 1 (mouth and facial movement), stage 2 (head nodding), stage 3 (forelimb clonus), stage 4 (rearing with forelimb clonus), and stage 5 (rearing and falling with forelimb clonus) (64). Animals that displayed no SRS were included for analysis in the early post-SE phase, whereas animals that exhibited two or more SRS with severity scores equal or greater than 3 were chosen for analysis in the epileptic phase. Animals were euthanized 7 days after SE for the early post-SE phase analysis, and 11 weeks after SE for the epileptic phase analysis. During the video-recordings, we observed a total of 80 SRS. Of these, 48.5% were classified as stage 5 according to the Racine Scale severity, 26.25% as stage 4, 18.75% as stage 3, 5% as stage 2, and 1.25% as stage 1. The average frequencies of different stages of spontaneous seizures recorded per animal in the epileptic group were 1.3 (stage 5), 0.7 (stage 4), 0.54 (stage 3), and 0.1 (stage 2). Only the animals which presented at least two stage 3 or higher seizures were included in the epileptic group.

### SLA

The rats were habituated individually to 37 × 24.2 × 24 cm cages for 14 days, and SLA was then continuously detected over a 10-day period (named as the baseline). The infrared motion sensors detect any movement inside the cage. They were placed 15 cm above the cage lids and automatically recorded the movement time in a computer every 5 min by the SAP System (Dr. Marconi Camara Rodrigues, Universidade Federal do Rio Grande do Norte, Natal, Brazil, 2011). After 240 h of baseline recordings, rats were subjected to SE. SLAs were again recorded from each animal for 10 days, starting both at 7 days post-SE (called early post-SE group) and at 10 weeks post-SE (epileptic group).

### RNA extraction and reverse transcription

The entire hippocampus was rapidly dissected, isolated on an ice-chilled plate and immediately frozen and stored in liquid nitrogen until RNA extraction. Total RNA was purified using Trizol reagent (Invitrogen, CA, USA) following the manufacturer's protocol. Total RNA from the left hippocampus of each rat was treated with DNase I (Ambion, TX, USA) for 30 min to avoid amplification of genomic DNA. Total RNA (1 μg) was reverse transcribed to single-stranded cDNA using the High-Capacity cDNA Reverse Transcription Kit (Applied Biosystems, Foster City, CA) according to manufacturer's instructions. Once reverse-transcription was complete, samples were diluted (10X) in TE (Tris 10 mM, pH 7.4; EDTA 0.1 mM, pH 8.0) and stored at –80°C until further analysis.

### qPCR

qPCR was carried out on a StepOnePlus PCR System (Applied Biosystems). The clock primer sequences and characteristics are described in previous reports (61, 65). Reactions were performed in a 12 μL volume containing cDNA (2 μL), 0.2 ± 0.6 μM each of specific forward (F) and reverse (R) primers, and 6 μl Power Syber1Green PCR Master Mix (Applied Biosystem, CA, USA). The amplification protocol was as follows: initial 10 min denaturation and 40 cycles of 95°C for 15 s and 60°C for 1 min. To ensure specificity of the PCR amplicon, we performed a melting curve analysis, ranging from 60 to 95°C, with temperature increases in steps of 0.5°C every 10 s. All primers showed an RT-qPCR efficiency ranging from 90 to 110%, as assessed by a standard curve based on a 5 points serial dilution of pooled cDNA (1:20; 1:40; 1:80; 1:160, and 1:320). The absence of contamination was confirmed using a no template control (NTC), with water in place of cDNA in each. Each assay was performed in triplicate, and the mean values were used for further analysis. The target gene expression was normalized to the *Tubb2a/Rplp1* as previously determined in our previous study as the best combination of reference genes for diurnal expression analysis in the hippocampus (61). A similar profile was seen with Tubb2a/Rplp1 in the prior study. Relative amounts of transcripts were calculated using the ^2^-ΔΔCt method (66). Values were expressed in quantities relative to the calibrator, which was run on each PCR plate through the entire experiment.

### Statistical analysis

The serial analysis of the locomotor activity was carried out using the software El Temps (Dr. Antoni Diez-Noguera, University of Barcelona, Barcelona, Spain, 1999). This software calculates all the rhythmic parameters, such as the period, onset, offset, alpha (active phase), acrophase, amplitude, the center of gravity, area under curve, mean waveform, and intracycle variability. The variables' comparisons between conditions were made by repeated measures ANOVA, followed by Bonferroni's *post hoc* tests.

Acrophase software (http://www.circadian.org/softwar.html) was used for the analysis of circadian patterns of clock genes expression. A *p* < 0.05 was taken as indicative of the presence of a rhythm with the 24 h (anticipated) period. Acrophase (ACRO) performs a cosinor regression to fit the time-course data to a cosine function that occurs in a 24 h period. The program detects periodicity and computes the achophase of circadian rhythm with the 95% confidence interval.

For gene expression's rhythm comparisons, the Spearman correlation and the cross-correlations were done using the IBM SPSS Statistics® (version 21). We considered high correlation when coefficient values were above 0.80, moderately correlation when values ranged from 0.50 −0.79, and low correlation when coefficient values were below 0.50. All datasets were assessed for normal distribution by Kolmogorov-Smirnov test. To compare mean gene expression per condition (the mean of all ZTs), we performed the Kruskal-Wallis test with Dunn's *post hoc* tests, once the distribution was found to be not normal according to the Kolmogorov-Smirnov test. Mean differences were considered statistically significant when *p* < 0.05, and the results were presented graphically as the mean and standard error of the mean.

## Results

### SLA

**Figure 2:**
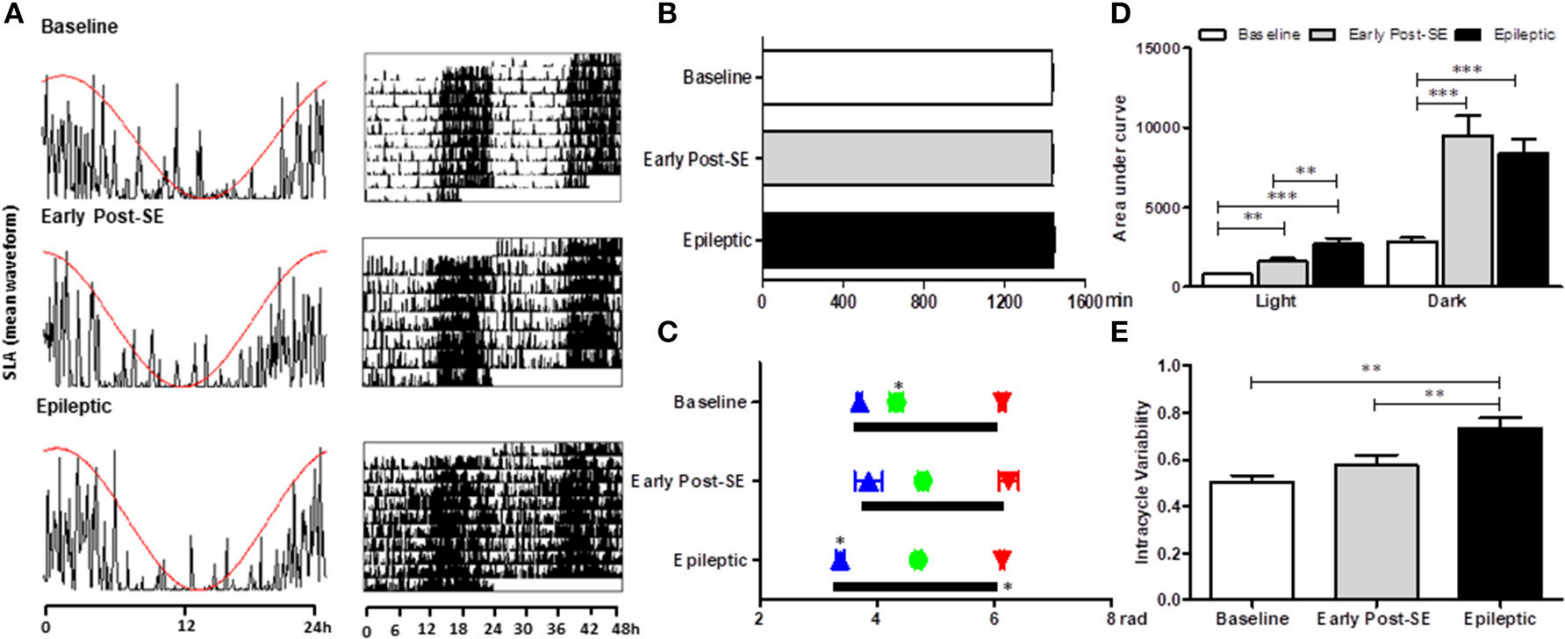
Circadian rhythm analysis of locomotor activity variables in different stages after SE induction. **(A)** Representative graphics of one-day activity waveform (left) with the cosine fit curve (red) and a representative actogram (right) in each condition. All three phases maintained the circadian rhythm of the rest-activity cycle, without alterations in the period **(B)**. **(C)** The activity onset (blue triangles) is similar to baseline rats and the ones in the early post-SE phase. However, the epileptic animals started the activity earlier than the others, meaning that the phase angle between the activity onset and the lights off is shorter in the epileptic condition. The activity's end time is similar for all 3 phases (red triangles). The acrophase (green balls) occurs later in the experimental (early post-SE and epileptic) groups. The duration of the activity phase (alpha, black bars) of epileptic animals is longer. **(D)** The amount of activity measured by the area under the curve of the mean waveform showed that the rats are less active during the light than during the dark in all three conditions. Besides, the experimental animals (early post-SE and epileptic) have higher locomotor activity both during the light and during the darkness. The activity of the epileptic rats is even higher than in the early post-SE condition during the light phase. **(E)** The epileptic condition has a more fragmented rhythm, represented by the higher intra-daily variability values. Statistical test for circadian analysis was done by Acrophase software and El Temps. Comparisons between conditions were done by repeated measures ANOVA, followed by Bonferroni's *post hoc* test. ^*^p < 0.05, ^**^p < 0.01, and ^***^p < 0.001.

All animals maintained the circadian rhythmicity of the SLA, with a similar period from the baseline [Figures 2A,B; Mean = 1437 ± 6 min; *F*_(2, 8)_ = 2.869, *p* = 0.115]. The SLA (area under the curve of the mean waveform) was higher in both early post-SE and epileptic phases. However, during the light period, epileptic animals presented an even more intense activity than the animals in the early post-SE phase [Figure 2D; *F*_(2, 8)_ = 18.954, *p* = 0.001; *post-hoc* Bonferroni *p* = 0.008 and *p* < 0.001]. Concerning the circadian phase (Figure 2C), the activity onset occurred earlier in epileptic rats [*F*_(2, 8)_ = 23.581, *p* < 0.001, *post-hoc* Bonferroni *p* < 0.001], compared to other conditions. However, the acrophase was observed later in epileptic animals in comparison to the baseline only. There was no difference in the activity's end time among conditions. The intracycle variabilities were higher in the epileptic phase compared to other conditions, revealing that the circadian rhythm of the locomotor activity is more fragmented in this condition [*F*_(2, 8)_ = 13.813, *p* = 0.003; *post-hoc* Bonferroni *p* = 0.003 and *p* = 0.005] (Figure 2E).

### Temporal profiling of clock transcript levels in post-SE model of MTLE

**Figure 3:**
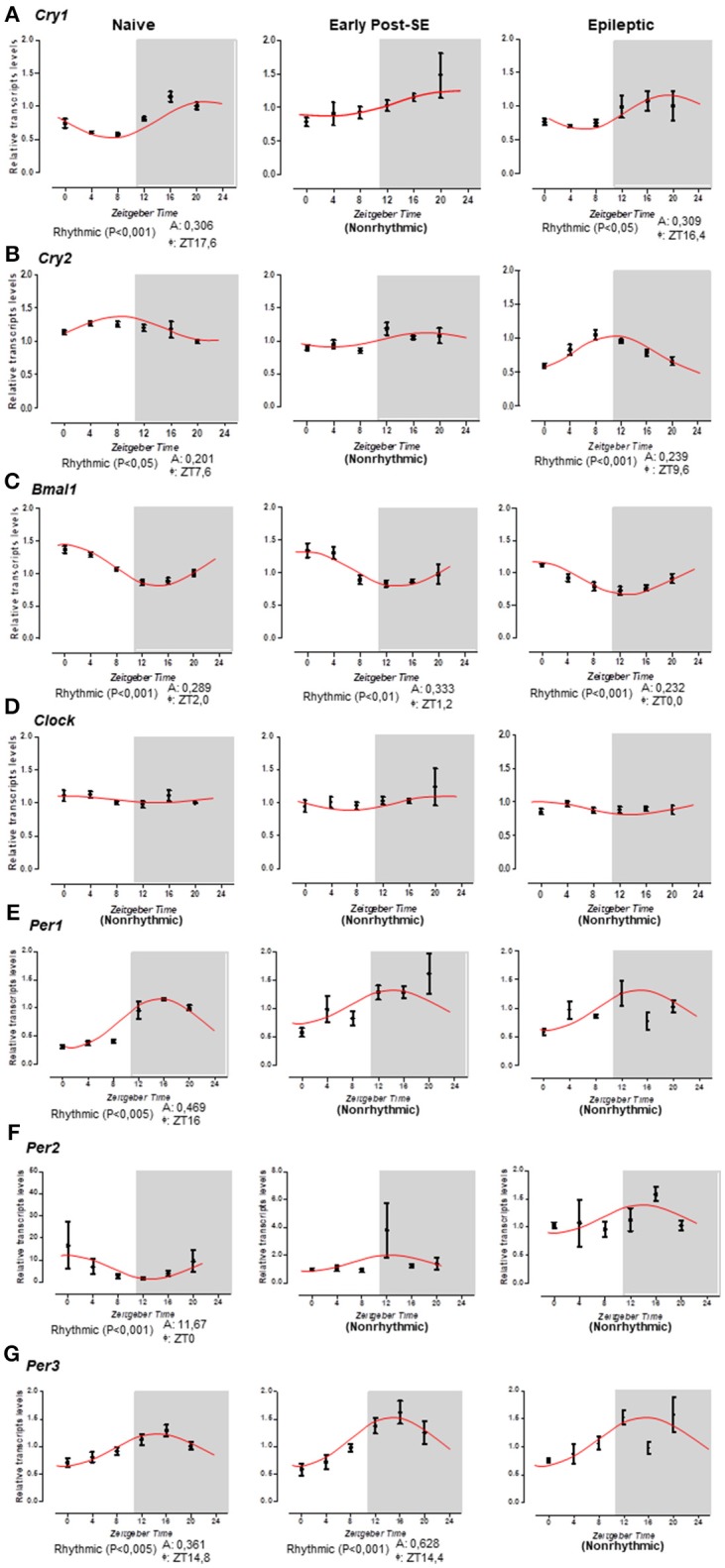
Temporal pattern of gene expression in the rat hippocampus. Relative transcripts levels for 6 time-points (ZT0, ZT4, ZT8, ZT12, ZT16, and ZT20) after normalization with *Tubb2a*/*Rplp1*. **(A)** The cosine curve adjusted for scattering points of *Cry1* relative gene expression revealed the presence of the rhythmicity in naïve (acrophase at ZT17.6, *p* < 0.001) and in epileptic conditions (acrophase at 16.4, *p* < 0.05), but the points in the early post-SE condition do not fit in a cosine curve, indicating no rhythmic expression throughout 24 h. **(B)** The same occurs for the *Cry2* gene; the relative gene expression showed the presence of the rhythmicity in naïve (acrophase at ZT17.6, *p* < 0.05) and in epileptic conditions (acrophase at 9.6, *p* < 0.001), but during the early post-SE, there is no rhythmic expression throughout 24 h. **(C)** *Bmal1* exhibits rhythmicity for all conditions—naïve, early post-SE and epileptic, although the acrophase phase shifted (acrophase at ZT2.0, *p* < 0.001, ZT1.2, *p* < 0.01, and ZT0, *p* < 0.001, respectively). **(D)** The *Clock* gene does not fit a cosine curve in any condition, revealing no circadian rhythmic expression. **(E)** For *Per1* gene, the relative gene expression showed the presence of the rhythmicity only in naïve (acrophase at ZT16, *p* < 0.005). **(F)** Relative gene expression for *Per2* gene showed the presence of the rhythmicity in naïve (acrophase at ZT0, *p* < 0.001). **(G)** *Per3* exhibit rhythmicity for naïve (acrophase at ZT14.8, *p* < 0.005) and early post-SE conditions (acrophase at ZT14.4, *p* < 0.001). Data are presented as mean ± SE per point. Statistical test for circadian analysis was done by Acrophase software.

We systematically evaluated the temporal profiling of *Bmal1, Clock, Cry1, Cry2, Per1, Per2*, and *Per3* mRNA levels in the hippocampus of naïve rats or rats subjected to SE (early post-SE and epileptic phases) sacrificed every 4 h during a 24 h period. Figure 3 illustrates the temporal organization and phase relationship of clock genes analyzed. Using the Acrophase software, we observed that only *Clock* did not show a rhythmic expression pattern in all conditions (Figure 3D). The oscillatory expression of *Bmal1* was kept in rats subjected to SE. However, the *Bmal1* acrophase was progressively phase advanced over epileptogenesis (ZT 1.2 for early post-SE condition and ZT 0 for epileptics) compared to naïve rats (ZT 2) (Figure 3C). Interestingly, the diurnal rhythm of *Cry1* and *Cry2* was lost in the early post-SE period but were restored in the epileptic condition with an earlier acrophase for *Cry1* (ZT16.4) and later for *Cry2* (ZT 9.6) compared to naïve rats (ZT 17.6 and ZT 7.6, respectively) (Figures 3A,B). However, by the ANOVA, a significant difference was observed for *Cry2* at a single time point. The *Per1, Per2*, and *Per3* expressions present circadian rhythms on the naïve condition, as expected (40, 67–69). However, the *Per1* and *Per2* rhythmic expressions were disrupted during the epileptogenesis, both during the early post-SE as well as the epileptic condition (Figures 3E,F). The *Per3* expression oscillates during the early post-SE, but the rhythm was lost in the epileptic group (Figure 3F).

*Bmal1* expression was in antiphase to *Per1 and Per3* in naïve (ZT 2 (*Bmal1*) x ZT 16 (*Per1*), rho = −0.829, *p* = 0.021 and ZT14.8 (*Per3*), rho = −0.946, *p* = 0.002), as well as to *Per3* in early post-SE (ZT 1.2 × 14.4, rho = −0.886, *p* = 0.009), and to *Cry2* in epileptic (ZT 0 × ZT 9.6, rho = −0.771, *p* = 0.036) rats, which is consistent with the negative temporal correlation found between these genes in these conditions (Figure 3). *Cry1, Cry2, Per1*, and *Per2* did not present a rhythmic profile in the cosinor analysis in the early post-SE period, which also showed high correlation with *Clock* [Cry1 rho = 0.886, *p* = 0.009 (Cry1); rho = 0.886, *p* = 0.009 (Cry2); rho = 0.943, *p* = 0.002 (Per1); rho = 0.886, *p* = 0.009 (Per2)]. *Cry2* became negatively correlated to *Bmal1* after SE (rho = −0.771, *p* = 0.036) probably due to the phase changes observed (Figures 3B,C).

**Figure 4:**
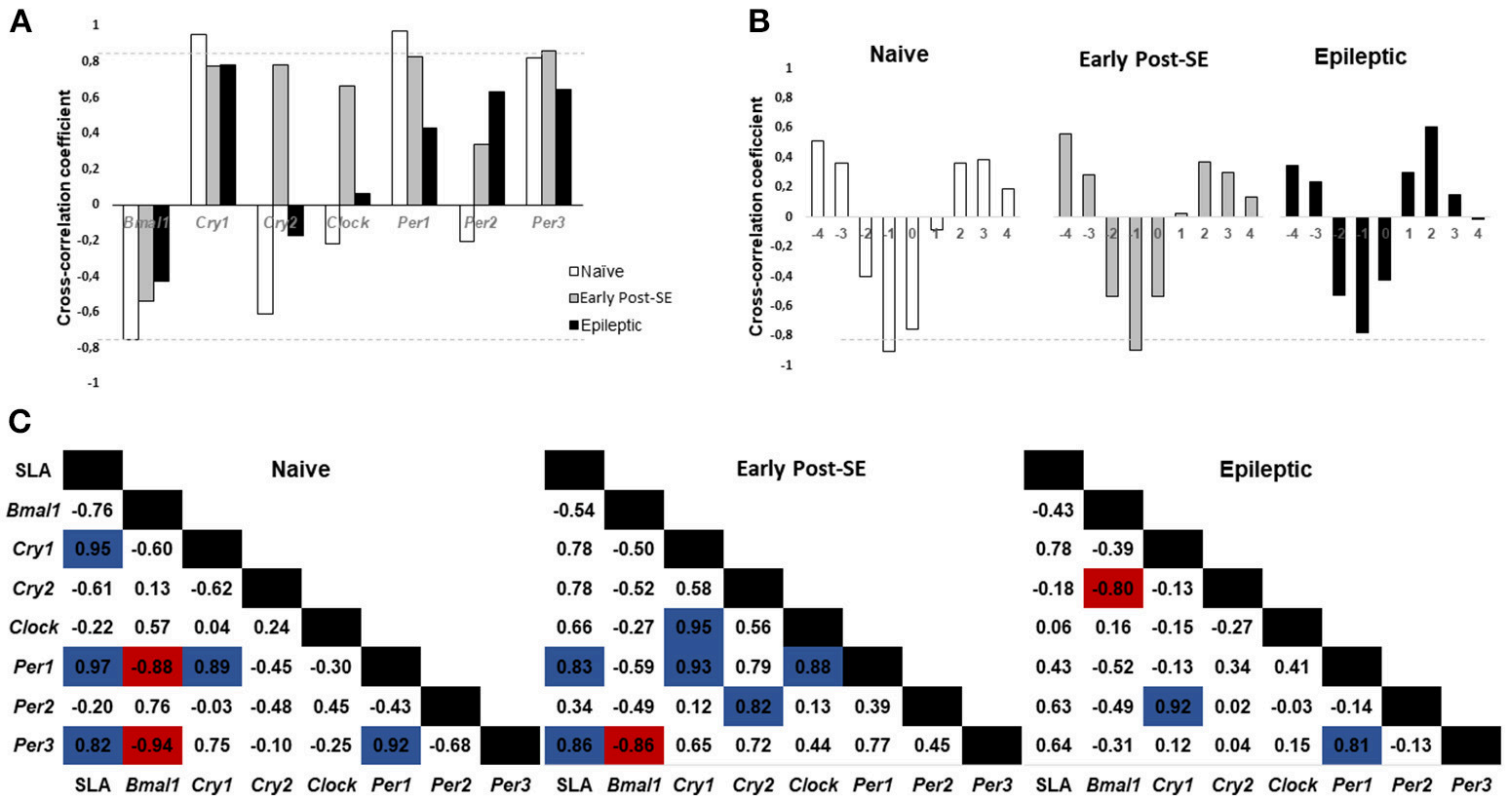
Cross-correlation analysis between SLA and gene expression. **(A)** Histogram of Spontaneous Locomotor Activity (SLA) rhythm cross-correlation coefficient at lag 0 versus gene expression. Coefficients above 0.80 (gray dotted line) are considered highly correlated. The SLA rhythm is highly correlated with *Cry1, Per1*, and *Per3* circadian gene expression in naïve rats and with *Per1* and *Per3* during the early post-SE. In epileptic rats, the SLA rhythm does not show high correlation with any gene expression rhythm. **(B)** Cross-correlation analysis between *Bmal1* expression and SLA in different lags. Note that the highest (negative) correlation occurs at lag −1 and it is higher than 0.80 for naïve and early post-SE condition, but not for the epileptic condition. **(C)** Cross-correlation coefficients for the SLA rhythm and all gene expression. The positive correlations are highlighted in blue, and the negative ones are in red.

We performed the cross-correlation analysis between the SLA and the expression of clock genes at lag 0 (Figure 4A). We found higher coefficient indices for *Cry1, Per1*, and *Per3* expression (0.95; 0.97, and 0.82, respectively). This pattern suggested that the rhythmic expression of these genes is highly correlated with the SLA rhythm in the naïve condition. This positive correlation is maintained during the early post-SE period for *Per1* and *Per3* (0.83 and 0.86) but decreased during the epileptic phase. On the other hand, *Bmal1* expression presented a moderate negative correlation with the SLA at lag 0. Verifying the cross-correlation at other lags, we found that at lag −1 the cross-correlation coefficient between *Bmal1* and SLA was higher, as shown in Figure 4B. At lag −1 the cross-correlation coefficient was −0.95 for naïve and −0.90 for the early post-SE group. The coefficient is reduced for the epileptic condition at lag −1 (−0.78), but increased at lag −2 and decreased at lag 0 when compared with the naïve (from −0.40 to −0.52 at lag −2 and from −0.76 to −0.425 at lag 0), indicating the phase shift observed throughout the epileptogenesis.

Finally, the cross-correlation between all the genes and the SLA are shown for each condition in Figure 4C. The naïve rats presented a positive correlation between the SLA and the *Cry1, Per1*, and *Per3* expression (0.95; 0.97, and 0.82, respectively). All these 4 components present the acrophase on the dark phase. There is a positive correlation between *Per1* and *Cry1* (0.89) and also between *Per1* and *Per3* (0.92) in this group. The *Bmal1* expression showed a negative correlation with the *Per1* and *Per3* expression (−0.88 and −0.94) since they are in antiphase (acrophases: *Bmal1* = ZT 2; *Per1* = ZT16 and *Per3* = ZT14.8) on the naïve group. In the early post-SE condition, some correlations were maintained, which include the SLA and *Per1* (0.83), SLA and *Per3* (0.86), *Cry1* and *Per1* (0.931), and *Bmal1* and *Per3* (−0.86). Additionally, there were three new correlations: *Cry1* and *Clock* (0.95), *Per1* and *Clock* (0.88) and *Per2* and *Cry2* (0.82). The association with *Clock* expression suggests the weakening of the rhythm, since *Clock* presents no rhythmic expression, as well as *Per2* and *Cry2* in the early post-SE period. Lastly, in the epileptic condition, the correlation between *Per1* and *Per3* returned (0.81). Despite losing the rhythm in this condition, the expression of these two genes presented the same pattern. The correlation between *Bmal1* and *Cry2* expression increased (−0.80), in as much as the *Cry2* rhythm return in antiphase with *Bmal1* expression (acrophases in ZT9.6 x ZT0). No high correlations were identified between gene expression in the hippocampus and SLA in the epileptic phase.

### Differential clock expression analysis in post-SE model of MTLE

**Figure 5:**
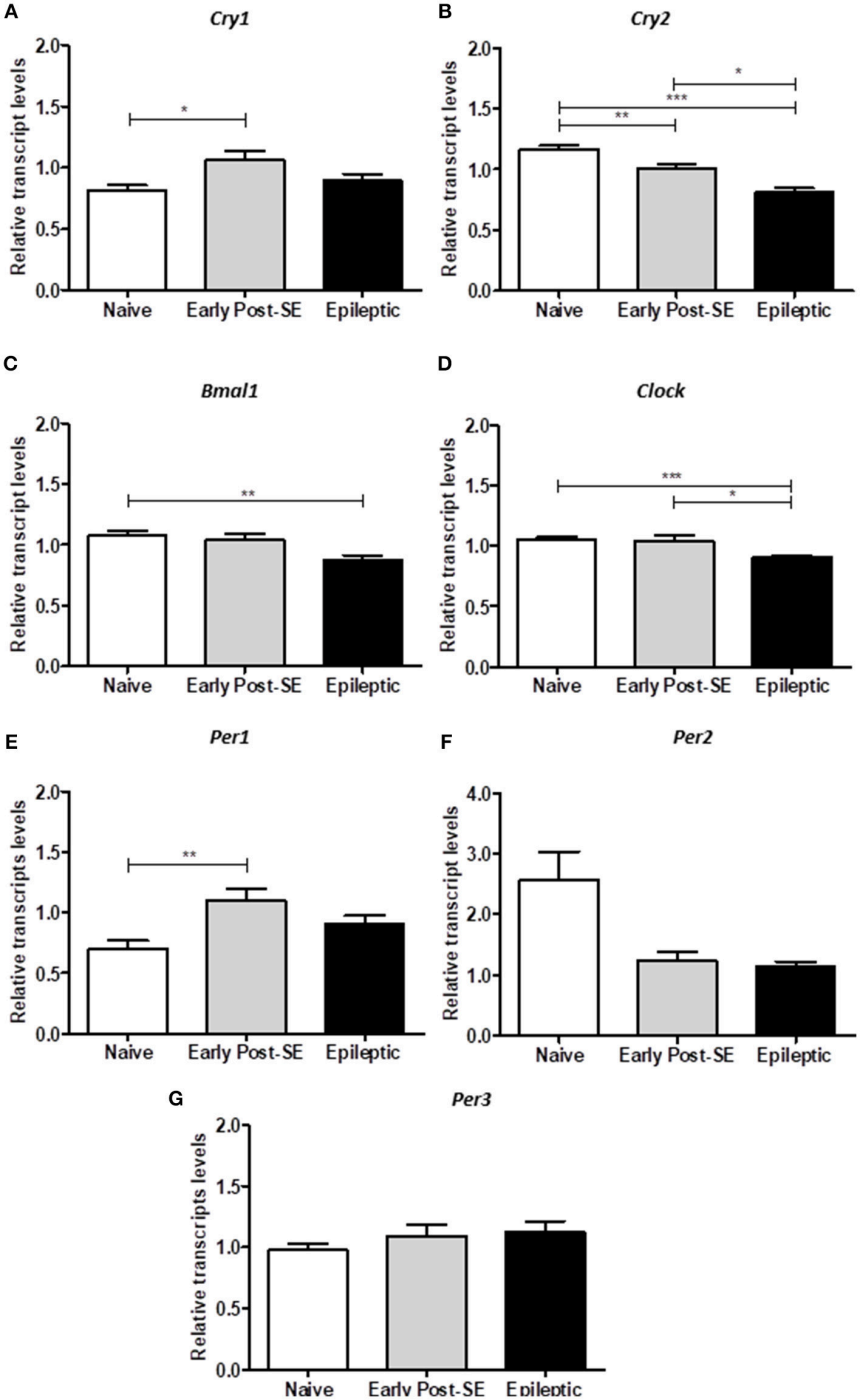
Differential gene expression in the post-SE model. The computed mean ± SE for each condition are represented in these histograms. **(A)** The mean of *Cry1* transcripts are increased in the early post-SE condition, compared to naïve, but returns to basal levels in the epileptic condition. **(B)** The *Cry2* expression is gradually decreased over the epileptogenesis. **(C)** The *Bmal1* relative transcript levels are diminished in the epileptic animals, compared to naïve. **(D)** The total *Clock* expression is similar in naïve and early post-SE phases, but is reduced in the epileptic condition. **(E)** The mean of *Per1* transcripts are increased in the early post-SE condition, compared to naïve, but returns to basal levels in the epileptic condition. No differences were found in *Per2* **(F)** and *Per3* **(G)** expression levels. One way ANOVA, followed by Bonferroni's *post hoc*. ^*^p < 0.05, ^**^p < 0.01, and ^***^p < 0.001.

To investigate if the overall expression of clock genes was dysregulated over SE-induced epileptogenesis in the hippocampus, we compared the transcript levels among epileptic, early post-SE and naïve conditions pooled from 6 different ZT (*n* = 5/ZT). The analysis of the mean gene expression (the mean from all ZTs) is required to verify the total gene expression variation throughout the epileptogenesis process, despite possible differences due to phase shifts. *Bmal1* and *Clock* transcripts were significantly decreased in the hippocampus of epileptic rats [H_(3)_ = 10.52, *p* = 0.0052, Figure 5C and H_(3)_ = 18.39, *p* = 0.0001, Figure 5D, respectively]. *Cry2* was progressively decreased over epileptogenesis [H_(3)_ = 32.39, *p* < 0.0001, Figure 5B], whereas *Cry1* and *Per1* increased in the early post-SE condition, and returned to normal levels in the epileptic condition [H_(3)_ = 7.808, *p* = 0.0202, Figure 5A and H_(3)_ = 10.96, *p* = 0.0042, Figure 5E, respectively]. *Per3* and *Per2* did not present significant differences among the groups (Figure 5).

## Discussion

### SLA

Considering that a few studies have examined the impact of SE on biological rhythms, our first objective was to evaluate the temporal pattern of SLA during epileptogenesis. Corroborating with the literature (56), we observed that: (i) the 24 h-oscillating SLA remains intact in post-SE groups, although the phase and the activity's amount change in early post-SE and epileptic conditions. (ii) Both light and dark phase SLA were increased after SE and remained elevated in the epileptic condition. (iii) The acrophase of the SLA rhythm was delayed during epileptogenesis. (iv) The SLA onset occurred earlier in epileptic animals. Our current findings complement these data by showing that epileptic rats present a more fragmented 24 h rhythmicity for SLA besides having an extended active phase (alpha) length and higher intracycle variability index, which was evidenced by a significant advance in activity onset followed by no changes on the activity's end time. Since they started the SLA earlier with a delayed acrophase, there was also a change in the phase angle.

It has been well documented that SE or limbic seizures do not abort the diurnal rhythm of SLA (27, 56), body temperature (57), and the EEG pattern (59). The EEG pattern is only transiently suppressed during a few days (2.9 ± 0.5 days) after PILO-induced SE (59). Besides, the phase shift (~12 h) observed in the hippocampus electrical activity does not alter the 24 h period of the global rhythms in epileptic animals, including the core body temperature and theta state transitions (70). Our observation that both SE and SRS do not abolish the rhythmic oscillation of some core clock genes, such as *Bmal1, Cry*1, and *Cry2*, provides molecular support for the maintenance of circadian output during the epileptogenic processes. Indeed, the loss of *Bmal1* in mice results in SLA impairment, reducing the activity level and abolishing the circadian rhythmicity in constant darkness (71).

On the other hand, in TLE, some aspects of the rhythms are altered, as evidenced by the advances and delays in circadian rhythm temperature (CRT) (58) and the long-term changes in the diurnal SLA rhythm. Since the hippocampus is an important peripheral oscillator, it is possible that the remapping in the hippocampus circadian outputs in epileptic rats contribute to modulation of some diurnal rhythms. In fact, the phase advance of SLA may be related to the phase advance observed for *Bmal1* in the hippocampus. Also, astrocytes in conditional *Bmal1* knockout mice showed a significantly advanced SLA onset after constant darkness (72), which may correlate to the reduced levels observed for *Bmal1* in the hippocampus in our study, concomitant to an earlier SLA onset. The disrupted expression of *Per1, Per2*, and *Per3* found in the epileptic group and the rearrangements in the correlation patterns during epileptogenesis could also be related to the modifications observed for SLA during this condition, such as a more fragmented activity. In this regard, the lack of significant correlations between SLA and gene expression in the epileptic group may indicate an uncoupling process between peripheral, and the central oscillators in this condition occurred during the neuronal rearrangement of the early post-SE period.

The contribution of alterations in the hippocampus to SLA is likely linked to the activation of hippocampal–accumbens pathway after PILO-SE induction as demonstrated previously (56). Alternatively, the SRS could modulate SCN-related functions (73). Notwithstanding, it has been shown that there is no histological damage at the SCN, in the PILO-model of epilepsy (56), though there is no physiological or molecular rhythm study to provide the evaluation about the temporal cellular activity inside the SCN in this model. The causality of these associations, however, cannot be determined herein. Also, a higher temporal resolution in data series would be necessary to precise changes in phase advance/delay between oscillators.

### Clock genes in early post-SE and epileptic phases

The current study describes, for the first time, the effect of SE on the temporal transcriptional expression of seven core clock genes in post-*Status Epilepticus*-model of mTLE. A remarkable feature in clinical and experimental TLE is that the SRS follows a 24 h periodicity and the pattern persists in a constant darkness condition (27). However, the molecular mechanism underlying this phenomenon is yet to be uncovered (74). We observed that the 24 h rhythmicity of *Bmal1, Cry1*, and *Cry2* transcripts in the hippocampus is maintained in epileptic animals, although with phase changes. However, we did not identify rhythmic expression for *Per1, Per2*, and *Per3* in epileptic rats. We also confirmed our previous finding that the *Clock* gene expression in the hippocampus of naïve rats does not fit in a cosine curve (61). This noncyclic expression pattern is kept in early post-SE and epileptic conditions. Since rhythmic expression of core clock proteins following the transcription of clock genes is fundamental to sustain the Transcriptional Translational Feedback Loops (TTFLs), it is expected that clock proteins also present rhythmicity in our samples when the mRNAs are rhythmic. The activity of these clock genes in the normal hippocampus may be regulating morphological and physiological circadian changes in this structure, including dendritic patterning and spine density, neurogenesis, and long-term potentiation (75–79). On the other hand, the circadian products oscillation in an epileptic hippocampus may contribute to rhythmic variation in the epilepsy threshold, and generation of SRS in a temporal pattern. In fact, it has been shown that the daytime or dark/light conditions influence the susceptibility for seizure onset and severity (51–53, 80) as well as the anticonvulsant efficacy of antiepileptic drugs (81). Thus, the oscillatory gene can drive a circadian availability of molecular factors that act in the electrical activity of the brain, resulting in rhythmic fluctuation in neuronal excitability (23, 82). Accordingly, a recent study using large-scale approach, demonstrated a diurnal remapping of the circadian molecular landscape in the hippocampus of a mouse model of TLE [(60), preprint publication]. Interestingly, *mTOR* pathway activity which is increased in epileptic animals demonstrates circadian oscillations (40, 83–86). Hepatic mTORC1 controls the daily levels of locomotor activity, body temperature, and lipid metabolism (84) and also show circadian activity (85–87). Furthermore, mTORC1 rhythmicity is controlled by the circadian molecular clock (85, 86). *Bmal1* negatively regulates mTORC1 signaling over a 24 h cycle by affecting the expression of *mtor* and *deptor* in the liver (86). An in-depth analysis of the temporal expression of these genes in the epileptic hippocampus and their potential time-dependent functional roles in the generation/maintenance of SRS in mTLE will be needed to comprehend better the function of these genes.

Regarding the pathogenesis of mTLE, SE-induced epileptogenesis cause alterations in several biological processes, such as gene expression, cell proliferation, dendritic plasticity, which are physiologically modulated by an endogenous circadian system (76, 77, 88). The current opinion is that these changes are related to compensatory mechanisms against the insult and/or to a series of epileptogenic processes that establish an epileptic state. In this study, we did not identify diurnal variation of *Cry1, Cry*2, *Per1*, and *Per2* mRNA expression during the early post-SE, whereas *Bmal1* presented an advanced acrophase. A phase advance was also demonstrated for *Cry1*, during the epileptic condition, while *Cry2* presented a phase delay, suggesting a dysregulation in the rhythm outputs of the biological processes occurring in this period. The confirmation and the consequences of this dysregulation on the epileptogenesis remain to be investigated. On the other hand, it is plausible that these oscillatory genes play functional roles in epileptogenic processes that are independent of circadian function. Indeed, CRY1 and CRY2 interact with nuclear receptors and modulate transcriptional activity (89). From this perspective, it is possible that diminished expression of *Cry2* and elevated expression of *Cry1* in the early post-SE phase may be involved in epileptogenicity, possibly due to its role in gene expression regulation. It is known that the epilepsy induced by pilocarpine reduces RORα mRNA and protein expression in the hippocampus of rats during the early post-SE phase (90). This variation can be the responsible for the *Cry*s rhythm expression's change during the early post-SE phase. Regarding *Cry1*, studies have shown that its functional role as a negative regulator of inflammatory processes (91). The reduced expression of *Cry1* key transcriptional targets lengthens the period of circadian molecular rhythms (92). In fact, the deletion of *Cry1* increases various proinflammatory cytokines, including IL-1β and TNF-α, through the NF-kB pathway (93). Furthermore, the overexpression of *Cry1* decreased the activation of TNF-α gene expression (94). Considering these, and the fact that TNF-α and other cytokines are elevated after SE, it is possible that the *Cry1* over-expression in the early post-SE condition is a compensatory anti-inflammatory activity. *Per1* up-regulation has also been seen upon depolarization *in vitro* or in epilepsy animal models, but its functional significance on epileptogenic process remains unclear (95). Thus, the exact role of the circadian oscillatory genes in the hippocampus remains unknown and functional assays will be important to disentangle the involvement of these clock genes on generation or maintenance of seizures.

In summary, the current study provides a systematic characterization of the temporal expression of *Bmal1, Clock, Cry1, Cry2, Per1, Per2, and Per3* transcripts and the SLA during SE-induced epileptogenesis. Our findings showed that although SLA circadian cycle is preserved in early post-SE and epileptic conditions, its circadian phase is changed and the rhythms were more fragmented in epileptic rats, suggesting that the phase coupling between the central and peripheral oscillators gets impaired by SRS. Additionally, we observed that *Bmal1* oscillation is intact in the early post-SE and epileptic phases, although with a phase advanced acrophase in the epileptic condition. *Cry1* and *Cry2* circadian oscillations were not detected in the early post-SE condition, which might be due to hippocampal reorganization. Nonetheless, the rhythmicity returns in the epileptic condition, also with phase shifts. These changes support the oscillation pattern of biological outputs emerged from the hippocampus after SE, including the 24 h periodicity in seizures. *Per1, Per2*, and *Per3*, however, lose the circadian rhythmicity in epileptic animals, aggravating the uncoupling between the oscillator and may contribute to epileptogenic processes independent of their circadian function. In general, the expressions of clock genes were significantly dysregulated during SE-induced epileptogenesis. There are some controversies regarding the relevance of the PILO-induced SE to human TLE (96, 97). Nonetheless, this animal prototype is widely used as it replicates the progression of multiple events observed in TLE. Hence, the findings of this animal model study are relevant for understanding the role of clock genes in epileptogenic processes and the development of chronic epilepsy after SE. Therefore, performing functional assays either by modulating the expression of the core clock genes in different stages of epileptogenesis or by inducing epilepsy in animals with a knockout of specific clock genes will be of great interest in future studies for addressing the specific role of individual clock genes epileptogenic processes.

## Author contributions

HM, BK, WP, and DG performed the experiments and the analysis. DG, OC, and MD designed the experiments. DG, AS, BK, TdA, JL, and MK wrote the article.

### Conflict of interest statement

The authors declare that the research was conducted in the absence of any commercial or financial relationships that could be construed as a potential conflict of interest.
